# Oxic Subseafloor Microbial Communities Retain Anabolic Capacity under both Oxic and Oxygen-depleted Conditions

**DOI:** 10.1264/jsme2.ME26008

**Published:** 2026-07-30

**Authors:** Mako Takada, Motoo Ito, Shu Ying Wee, Jason B. Sylvan, Rosalind M. Coggon, Emily R. Estes, William P. Gilhooly III, Yi Wang, Masataka Aizawa, Susumu Yoshizawa, Yuki Morono

**Affiliations:** 1 Atmosphere and Ocean Research Institute, The University of Tokyo, Japan; 2 Graduate School of Frontier Sciences, The University of Tokyo, Japan; 3 Kochi Institute for Core Sample Research, Japan Agency for Marine-Earth Science and Technology (JAMSTEC), Japan; 4 University of Southern California, USA; 5 Texas A&M University, USA; 6 University of Southampton, United Kingdom; 7 Now at National Science Foundation, Alexandria, VA, USA; 8 Indiana University Indianapolis, USA; 9 Tulane University, USA; 10 Hokkaido University of Education, Japan

**Keywords:** subseafloor biosphere, anabolic metabolism, stable isotope incubation, NanoSIMS, single-cell isotope imaging

## Abstract

Marine subseafloor sediments underlying open ocean regions are predominantly low-energy environments; however, they harbor vast microbial populations. How these communities persist and whether they retain anabolic capacity during transient oxygen depletion remain unclear. We herein investigated oxic sediments from the South Atlantic Gyre (SAG; IODP Site U1559D, core 7X-1) using paired incubations under oxic and anoxic (oxygen-depleted) conditions with defined C/N amendments. Sediments were amended with a ^13^C-amino acid mix+^15^N-ammonium, ^13^C-bicarbonate+^15^N-ammonium, or ^15^N-ammonium only and then incubated for 10, 30, and 75 d. Single-cell nanoscale secondary ion mass spectrometry (NanoSIMS) confirmed the assimilation of isotope-labeled substrates under oxic and anoxic conditions. Pronounced ^13^C enrichment occurred primarily in amino acid-amended incubations, whereas ^15^N incorporation from ammonium was widespread across all treatments, including ammonium-only incubations without added organic carbon, indicating assimilation supported by endogenous sedimentary carbon pools. Biomass-based growth responses varied among amendments and redox regimes, demonstrating that oxic-sourced communities may transition into measurable growth even under oxygen depletion. Amplicon-based community profiles exhibited directional, time-dependent shifts with limited segregation by substrate identity,‍ ‍suggesting that incubation time and shared successional dynamics dominated over amendment identity at the whole-community level. Collectively, these results indicate that SAG subseafloor communities retain a flexible anabolic potential that may be expressed under both oxic and oxygen-depleted conditions when substrates and/or oxidants become available.

Marine sediments cover ~70% of Earth’s surface and host vast microbial communities that account for 12–45% of Earth’s microbial cells or ~0.6–2% of the total living biomass (Gt-C) (Whitman *et al.*, 1998; Kallmeyer *et al.*, 2012; Bar-On *et al.*, 2018; Magnabosco *et al.*, 2018; Morono *et al.*, 2020). Global surveys revealed high taxonomic breadth and clear biogeographic zonation in subseafloor microbiomes (Hoshino *et al.*, 2020). Cell concentrations generally decline from continental margins toward the oligotrophic gyres (Kallmeyer *et al.*, 2012). In many ultra-oligotrophic settings, dissolved oxygen may penetrate meters below the seafloor—and all the way to the basaltic basement in some provinces—indicating chronically low respiration rates (D’Hondt *et al.*, 2015). Under such extreme energy limitation, synthesis studies suggest that maintenance metabolism dominates, turnover times stretch from centuries to millennia, and survival strategies, such as dormancy, spore formation, and slow anabolism, prevail (Lever *et al.*, 2015; Bradley *et al.*, 2020). After burial, cell motility is strongly constrained by the compacted, fine-grained sediment matrix; in contrast, dissolved oxidants and substrates are transported by diffusion, while temperature, pressure, and redox conditions gradually evolve over geological timescales. Consequently, communities may reflect depositional conditions and survive long after burial (Orsi, 2018). High-resolution records also show that subsets of taxa may retain a depth-independent paleoenvironmental signal under relatively weak post-burial selection, such as in Arabian Sea sapropel sequences (Orsi *et al.*, 2017). At the same time, limited motility and long exposure histories imply that communities may progressively adapt to ambient redox regimes (Morono *et al.*, 2020).

Cumulative evidence demonstrates that subseafloor microbes are alive and, in some settings, metabolically responsive. Depositional history may imprint the community structure; however, anaerobic lineages may persist even in nominally oxic settings via microscale anoxic niches and local oxygen scavenging (Jalaluddin *et al.*, 2025). Conversely, in the South Pacific Gyre (SPG), very old (~100‍ ‍Ma) oxic sediments showed growth only under oxic incubations and no activity under anoxia, which is consistent with long-term redox imprinting (Morono *et al.*, 2020).

The South Atlantic Gyre (SAG) offers a critical test bed for deep-biosphere ecology because oxygen penetration is predicted to be deep under the region’s extremely low sedimentation and supply of organic matter. At IODP Site U1559, pore water dissolved oxygen measurements were conducted throughout the sediment column, revealing a mid-depth anoxic interval and detectable oxygen near the sediment–basement interface—consistent with oxygen consumption within the sediment and re-introduction via oxygenated basement fluids. We herein focused on oligotrophic SAG sediments from Hole U1559D, Core 7X-1 (49.9 meters below seafloor [mbsf] and 1.1 meters above basement [Coggon *et al.*, 2024b]), which likely experienced a sustained oxic regime over ~6 Myr. We investigated whether buried communities retained anabolic capacity and how oxygen availability and substrate quality modulated responses. We hypothesized that buried SAG communities may retain anabolic capacity under both oxic and anoxic incubations, with oxygen exerting weaker control after a shorter oxic burial history (~6 Myr) than previously reported for ~100-Ma SPG sediments. To test this hypothesis, we conducted time-course incubations (10, 30, and 75 d) with stable isotope-labeled substrates (^13^C-amino acid mix + ^15^N-ammonium, ^13^C-bicarbonate + ^15^N-ammonium, and ^15^N-ammonium alone) under oxic and anoxic conditions. We quantified per-cell isotope assimilation using nanoscale secondary ion mass spectrometry (NanoSIMS), measured biomass changes by cell counts, and profiled microbial communities. By linking single-cell isotope uptake to population growth and community shifts, we elucidated how oxygen availability and substrate quality jointly constrain anabolic potential in long-buried oligotrophic sediments. By pairing NanoSIMS measurements with fluorescence-activated cell sorting (FACS) and 16S rRNA gene profiling from sorted cells, we linked single-cell C/N assimilation to population-level growth and time-resolved community changes in an ultralow-biomass oxic sediment system. This integrated approach provides a quantitative framework to evaluate how substrate quality and redox conditions modulate anabolic responses in diffusion-dominated, energy-limited subseafloor habitats.

## Materials and Methods

### Samples

Sediment samples used in this study were collected during IODP Expedition 390 in the SAG. The samples collected from Site U1559 (30°15.6336'S, 15°02.0941'W) are silty clays and calcareous nannofossil oozes. Water depth at the site was 3,055 m below sea level. Whole-round core samples of U1559D 7X-1 (obtained from 49.9 mbsf) were used for incubation experiments as described in the following section. A drilling fluid contamination assessment by the perfluorocarbon tracer PFMD revealed minimal or no contamination during the coring process (Coggon *et al.*, 2024b).

### Porewater dissolved oxygen (Sites U1559C and U1559D)

Dissolved oxygen concentrations in interstitial water were measured shipboard on intact whole-round cores prior to splitting using PM-PSt7 oxygen profiling microsensors (PreSens, Regensburg), as described by Coggon *et al.* (2024a). Oxygen sensors were calibrated daily using air-saturated deionized water (100% oxygen saturation) and 10 g L^–1^ sodium sulfite (Na_2_SO_3_) solutions (0% oxygen saturation). Measurements were performed along the cores at an effective downhole resolution of ~1.5 m by drilling a hole in the core liner and sticking the probe into the sediment. To minimize atmospheric contamination, cores were kept capped and handled immediately after recovery. Temperature and salinity were recorded and entered for each measurement because oxygen solubility (and the optical sensor calibration) depends on these parameters; the instrument uses them to convert phase-shift signals to oxygen concentrations (Tengberg *et al.*, 2006).

### Incubation experiments

To identify autotrophic and heterotrophic microbial populations and estimate potential growth and substrate uptake rates, we incubated sediment samples with stable isotope-labeled substrates: ^13^C-bicarbonate (NaH^13^CO_3_; 99 atom% ^13^C; CIM-441-MTP), a mixture of ^13^C-labeled amino acids (16 amino acids: alanine, arginine, aspartic acid, glutamic acid, glycine, histidine, isoleucine, leucine, lysine, methionine, phenylalanine, proline, serine, threonine, tyrosine, and valine; 97–99 atom% ^13^C; CLM-1548-0), and ^15^N-ammonium (^15^NH_4_Cl; 99 atom% ^15^N; NLM-467-0) (Cambridge Isotope Laboratories). Incubations were initiated onboard during the expedition. To minimize potential contamination by drilling fluid, we subsampled the interior of intact cores using sterile, tip-cut 30-mL syringes; on average, seven mini-cores (15 cm^3^ each) were collected from each core and transferred into sterilized 50-‍mL glass vials (Nichidenrika-Glass) sealed with a sterile butyl rubber stopper and screw cap. Vials were flushed with 0.22-μm filtered N_2_ and stored at 4°C prior to substrate addition. All materials were sterilized by autoclaving at 121°C for 20 min. In oxic incubations, the vial headspace oxygen was adjusted to ~3.3% (v/v) by adding 0.22-μm filtered air. Consequently, oxic incubations contained ~53 μmol oxygen per vial and anoxic incubations contained ~1.4 μmol residual oxygen derived from pore water. Therefore, “anoxic” conditions here refer to oxygen-depleted, non-replenished systems rather than strictly oxygen-free conditions. Labeled substrates were injected through the septum as sterile aqueous stocks: 150 μL per substrate per vial (15 μmol of the relevant ^13^C-labeled substrate and/or 1.5 μmol of ^15^NH_4_Cl), and vials were incubated at 4°C. All reagents and gases, including air used for preparation, were passed through 0.22-μm syringe-top filters. Immediately after set-up, a split of the same sediment was fixed to define the initial time point (T_0_) by adding an equal volume (15 mL) of 4% paraformaldehyde (PFA) in PBS and holding at 4°C for 5 h. At subsequent time points (T_1_, ~10 d; T_2_, 30 d; T_3_, 75 d), vials were opened and sediments were fixed in the same manner as T_0_. Fixed samples were frozen at –80°C while being transferred to the onshore laboratory, thawed and washed twice with PBS, and then preserved in PBS/ethanol (1:1, v/v) at –20°C until analyzed. The size of the mini-core (15 cm^3^ each) per vial for the incubation was selected in consideration of available core materials (15-cm whole rounds, corresponding to 300 cm^3^ of interior intact cores) and the expected number of microbial cells in sediment samples (to ensure the detection of microbes even at a very low cell abundance [<10^3^ cells‍ ‍cm^–3^]). Consequently, each incubation condition/time point was represented by a single vial. Therefore, temporal patterns were interpreted descriptively rather than as statistical effects.

### Cell enumeration and selective sorting onto the membrane

To efficiently analyze substrate incorporation into microbial cells with NanoSIMS, cells were separated from their sediment matrix and FACS was conducted to concentrate and purify cells in a small area for anal­ysis (~0.5 mm^2^) (Morono *et al.*, 2013). To avoid any contamination, sample preparation, including cell separation and FACS, was conducted in clean rooms at the Kochi Institute for Core Sample Research, Japan Agency for Marine-Earth Science and Technology (JAMSTEC).

Cell separation, microscopy, and sorting procedures followed the method of Morono *et al.* (2020) with slight modifications. The start material was 1 mL of fixed slurry (1/3‍ ‍[v/v] sediment in ethanol-PBS solution), which was washed and resuspended in 2.5% NaCl solution to provide 3.2 mL of sediment slurry. Microbial cells in the sediment slurry were then separated, half of the separated cell suspension was trapped and stained by SYBR Green I, and cells in the suspension were concentrated to a volume of 0.5 mL by centrifugation. The other half of the separated cell suspension was filtered onto a black polycarbonate membrane with a pore size of 0.22 μm (GTBP02500; MilliporeSigma) and used to count microbial cells by the fluorescence color-based discriminative cell enumeration method (Morono *et al.*, 2009; Morono and Inagaki, 2010). Stained cells were directly sorted onto 0.2-μm polycarbonate membranes that were coated with indium tin oxide (ITO) (for the NanoSIMS anal­ysis) or uncoated (for DNA extraction, Isopore, GTBP02500; MilliporeSigma). ITO-coated membranes were prepared by sputter deposition at Astellatech. Sorted cells on uncoated membranes were stored at –20°C until DNA extraction.

### NanoSIMS anal­ysis of single cell-image acquisition and data processing

Cell targets were identified by the fluorescence of the SYBR Green I stain and marked on NanoSIMS membranes with a laser dissection microscope (LMD6000; Leica Microsystems) to facilitate rediscovery of the analysis spots during NanoSIMS analysis. Microbial cells that incorporated stable isotope-labeled substrates were analyzed using NanoSIMS 50L (CAMECA, AMETEK) at the Kochi Institute for Core Sample Research, JAMSTEC, Japan. Samples on the ITO-coated polycarbonate membrane were pre-sputtered at high beam currents (30 pA s^–1^ μm^–2^) before measurements. ^12^C^–^, ^13^C^–^, ^12^C^14^N^–^, ^12^C^15^N^–^, and ^32^S^–^ secondary ions were collected and measured in parallel at a mass resolution of 8000, which was sufficient to separate ^13^C^–^ from ^12^CH^–^ and ^12^C^15^N^–^ from ^13^C^14^N^–^. Samples were measured using a 1–2 pA Cs^+^ primary beam that was rastered 20 times over a 24×24 μm field of 256×256 pixels with a counting time of 3 ms per pixel. Recorded images and data were processed using the OpenMIMS plugin (Gormanns *et al.*, 2012) in ImageJ (Schneider *et al.*, 2012) with the Fiji distribution (Schindelin *et al.*, 2012). Different scans of each image were aligned to correct image drift during acquisition. Final images were created by adding the secondary ion counts of each recorded secondary ion from each pixel over all scans. Intracellular carbon and nitrogen uptake from stable isotope-labeled substrates was calculated by drawing regions of interest (ROIs) on CN^–^ images (recognizing cells in the images) and calculating ^13^C/^12^C and the ^15^N/^14^N ratio (calculated from the ^12^C^15^N/^12^C^14^N ratio). The concentrations of bicarbonate (DIC) and ammonium in the original sample measured on board (1.95 mM and 17 μM, respectively) were used to calculate the substrate incorporation ratio (atom%) for bicarbonate and ammonium in single cells.

### Substrate incorporation and rate calculations

Rates of microbial biomass synthesis were estimated following Morono *et al.* (2020) with minor adaptations to our dataset. We computed (i) the biomass-based specific growth rate *μ*_B_ (Eq. 1) from cell abundances at the start and end of each incubation (*X₀* and *Xt*), and (ii) the substrate incorporation–based biomass generation rates for carbon and nitrogen (^C^*μ*_S_ and ^N^*μ*_S_, Eqs. 2 and 3).

$$\mu_{B}=\frac{\ln X_{t}-\ln X_{0}}{t}\;(1)$$

μSC=(−ln⁡(1−(FtC−FnatC)(FlabelC−FnatC)))/t (2)

μSN=(−ln⁡(1−(FtN−FnatN)(FlabelN−FnatN)))/t (3)

Regarding ^C^*μ*_S_ and ^N^*μ*_S_, rates were derived from cellular isotope atom fractions measured by single-cell NanoSIMS, using incubation time *t*, labeling strength F_label_, measured atom fraction F_t_, and natural abundance F_nat_. To be conservative, we analyzed only “active” ROIs with ^13^C or ^15^N ratios that exceeded the background threshold defined from polycarbonate membranes (mean+3 SD), and assumed that C and N incorporated into newly synthesized biomass were entirely derived from the amended substrates. The estimated initial active fraction *f*_0_ was calculated from the observed active-ROI fraction ft and the biomass-increase factor A following Eqs. 4, 5, and 6.

$$X_{t}=A\,X_{0}\;(4)$$

$$X_{0}\,\left(1-f_{0}\right)=X_{t}\,\left(1-f_{t}\right)\left[f_{t}<1,X_{t}\,\left(1-f_{t}\right)<X_{0}\right]\;(5)$$

$$f_{0}=1-A\,\left(1-f_{t}\right)\;(6)$$

### DNA extraction and sequencing

Microbial cells sorted onto non-coated polycarbonate membranes were subjected to alkaline lysis in a clean-air facility for DNA extraction, library preparation, and amplicon sequencing, following the procedure modified from Morono *et al.* (2014, 2020). We sorted and trapped cells on a polycarbonate membrane by a vacuum to remove the sheath fluid that potentially contained contaminants (Goethals *et al.*, 2025). To minimize membrane-derived contamination, we excised a small spot (~1‍ ‍mmφ) of the membrane that had sorted cells by laser microdissection (LMD-6000; Leica Microsystems). Cell lysis was conducted using three sequential alkaline treatments with intermediate neutralization, and the combined lysate was used after concentration by ethanol precipitation as the template for downstream PCR amplification. Extraction-negative controls were processed using blank membranes treated identically to sample membranes. The V4 region of the 16S rRNA gene was amplified using the universal primers U515F and U806R (Caporaso *et al.*, 2012). To avoid overamplification, the number of PCR cycles was set between 25 and 40 based on the results of preliminary PCR tests with monitoring amplification curves. PCR no-template controls were included throughout amplification steps. Amplicons from the first-round PCR were gel-purified and subsequently quantified fluorometrically. A second round of PCR was performed to attach dual indices and sequencing adapters. Library quality was confirmed by fluorometric quantification and a fragment size distribution anal­ysis. Indexed libraries were sequenced on an Illumina NextSeq 1000 system (paired-end, 2×300 bp) using the NextSeq 1000/2000 P1 Reagents (600 cycles) at Bioengineering Lab. Sequence reads were processed using QIIME 2 (Bolyen *et al.*, 2019). Primer sequences were removed and low-quality bases were trimmed prior to denoising. Paired-end reads were quality filtered, merged, denoised, and checked for chimeras using the DADA2 plugin (Callahan *et al.*, 2016), generating amplicon sequence variants (ASVs). Negative-control samples were used to identify potential contaminants, and ASVs detected in negative controls were removed from the ASV pool used for downstream anal­yses. Representative ASV sequences were taxonomically assigned using the EzBioCloud 16S rRNA gene reference database. Downstream anal­yses were performed in R using ASV tables with the packages vegan (Dixon, 2003) and phyloseq (McMurdie and Holmes, 2013).

In non-metric multidimensional scaling (NMDS), we used centered log-ratio (CLR)-transformed ASV compositions. Since the ASV table is compositional, zeros were replaced with a small pseudocount prior to CLR transformation, and NMDS was performed on Aitchison distances (Euclidean distances in CLR space). CLR minimizes closure-induced distortions, is invariant to library-size differences, and avoids the information loss and stochasticity of rarefaction. NMDS based on rarefied counts and on untransformed relative abundances is provided for comparison in Fig. S1. Since 2D ordination stress was moderate (0.22), we interpreted patterns as gradients rather than discrete clusters. Accordingly, we presented the CLR-based ordination as the main figure, with alternative normalizations shown in Fig. S1.

## Results and Discussion

### Cell abundance and oxygen profiles in site U1559 sediments

We elucidated the depth profiles of cell abundance and dissolved oxygen in sediments from Holes U1559C and U1559D (Fig. 1). Cell abundance was consistently low, in the order of 10^4^ cells cm^–3^ at the U1559D 7X-1 horizon (1.79×10^4^ cells cm^–3^ at 49.9 mbsf). Dissolved oxygen declined from 157.4 μM at 0.5 mbsf in Hole U1559C and 119.0 μM at 0.9 mbsf in Hole U1559D to near-zero values by 12.7–12.6 mbsf (0.39 μM in U1559C and 0.37 μM in U1559D), indicating oxygen consumption in the shallow sediment column. Oxygen concentrations then increased again toward the basement, reaching 156.6 μM at 52.85 mbsf in Hole U1559C and 93.73 μM at 48.60 mbsf in Hole U1559D, suggesting oxygen penetration from the basaltic basement and the presence of a mid-depth anoxic zone. This low-biomass setting is consistent with the global relationship between the subseafloor biomass, TOC availability, and burial depth (Lipp *et al.*, 2008; Kallmeyer *et al.*, 2012). Deep oxygen penetration from the seafloor to approximately 10–13 mbsf suggests that these communities experienced strong energy limitations and likely persisted mainly via maintenance-level respiration over geological timescales (D’Hondt *et al.*, 2015).

We also exami­ned estimated oxygenation histories for sediment sample 7X-1 (49.9 mbsf and 1.1 meters above the basement). Since sample 7X-1 was located 1.1 m above the basement and the sediment was organic-poor and oligotrophic, the sediment at 7X-1 was likely oxygenated for much of its burial history. In parallel, cell densities decline with depth, and oxygen gradients in oligotrophic sediments are typically less steep than those in the uppermost seafloor sediments, where organic matter supply and respiration rates are the highest. This pattern implies low net oxygen consumption and extremely low cell-specific respiration, insufficient to deplete oxygen to anoxia within 1.1 m of the oxygen-supplying basement. Steeper oxygen gradients and the accumulation of reduced products (*e.g.*, Fe^2+^ and sulfide) were expected with high cell-specific respiration; however, these were not observed in any holes at Site U1559 (Coggon *et al.*, 2024b). Low but measurable Mn^2+^ levels were detected (Coggon *et al.*, 2024b), suggesting that Mn reductions occur in anoxic microniches within oxygenated sediments.

### Single-cell isotope assimilation and growth dynamics across substrate and oxygen conditions

To elucidate how subseafloor microbes assimilate carbon and nitrogen under different substrate and oxygen conditions, we performed single-cell NanoSIMS imaging after incubating sediment samples with ^13^C- and/or ^15^N-labeled substrates under oxic and anoxic conditions (Fig. 2). The total number of cellular ROIs analyzed was 801, ranging from 6 to 110 per incubation. ROI counts varied across incubations primarily due to differences in recoverable cell densities on membranes and the available NanoSIMS anal­ysis time; samples with a lower concentration of microbial cells yielded fewer analyzable ROIs. Cells incubated with a ^13^C-amino acid mix and ^15^N-ammonium showed increased ^13^C/^12^C and ^15^N/^14^N ratios, confirming concurrent carbon and nitrogen assimilation at the single-cell level (Fig. 2b and c). Conversely, in all treatments, cells with low or undetectable incorporation of substrates were observed, suggesting cell-to-cell heterogeneity arising from differences in the physiological state and substrate use (Fig. 2d). In treatments where ammonium chloride was added as the sole nitrogen source, increases in ^15^N/^14^N were also observed, indicating ongoing ammonium assimilation (Fig. 2g).

We quantified population and single-cell responses in time-course incubations. Cell numbers increased in all treatments, with the largest changes being observed in the amino acid mix+ammonium incubations—more pronounced under anoxic incubations—whereas increases under bicarbonate+ammonium were more gradual. In ammonium-only incubations, the addition of oxygen produced a greater increase in cell numbers (Fig. 3a). ROI-based NanoSIMS ratios paralleled these changes (Fig. 3b and c): pronounced ^13^C enrichment occurred only with amino acid mix amendments, while ^15^N enrichment was widespread across all conditions, including the ammonium-only amendment. Collectively, these patterns indicate that amino acid amendments uniquely supported the rapid uptake of added organic carbon (^13^C) and promoted coupled C–N assimilation at the single-cell level, whereas ammonium-only incubations showed that increasing ammonium availability may stimulate net growth even without added organic carbon.

Mechanistically, the amino acid mix+ammonium treatment provides highly accessible C and N, enabling fast assimilation and growth. Under the idealized assumption that amended substrates are the sole sources, the theoretical maximum labeled fraction after n doublings is (1–2^–n^). Observed enrichments in all conditions fell below this range (Fig. 3b and c), implying the incorporation of unlabeled pools, including sedimentary TOC (~0.17 wt%) and TN (~0.04 wt%). Depressurization to ambient laboratory conditions may have relaxed the compacted sediment matrix and improved microbial access to these endogenous pools (Estes *et al.*, 2019). With ammonium alone, cells must draw carbon skeletons from sedimentary TOC and/or dissolved inorganic carbon (DIC); ammonium incorporation may proceed via assimilatory pathways (*e.g.*, GS/GOGAT) that require endogenous carbon skeletons and reducing power.

Across substrate and redox regimes, growth and isotope-derived production metrics showed small but consistent differences (Table 1). Biomass-based specific growth rates (*μ*_B_) under ammonium-only incubations showed the largest *μ*_B_ values up to 0.315 d^–1^. Since pore water ammonium was ~17 μM and vials were amended to a final concentration of ~164 μM (including the background), this enrichment may have alleviated potential ammonium limitation during incubations, enabling rapid population-level growth supported by endogenous sedimentary carbon pools. In contrast, oxic amino acid mix+ammonium incubations reached moderate *μ*_B_ values of ~0.23 d^–1^ (doubling time T_d_ ~3 d). Therefore, the highest net biomass increase did not necessarily coincide with the addition of labile organic carbon, consistent with growth fueled by endogenous resources once key constraints (including ammonium availability and oxidant supply) are relaxed; nevertheless, amino acid additions clearly promoted coupled C–N assimilation, as evidenced by strong ^13^C enrichment.

Under anoxic conditions, microbial communities amended with an amino acid mix showed similar growth to that under an oxic incubation, whereas the strong growth response observed in oxic ammonium-only incubations (described above) was attenuated under anoxia. In bicarbonate+ammonium incubations, *μ*_B_ values were smaller but consistently positive, and carbon-based generation rates (^C^*μ*_S_) were insufficient to fully account for the measured increases in total cell abundance, indicating that bicarbonate-derived carbon was not the dominant source of biomass. Since bicarbonate provides inorganic carbon but not energy, substantial net CO_2_ fixation requires additional electron donors and sufficient reducing power (Fuchs, 2011; Hügler and Sievert, 2011); therefore, low ^C^*μ*_S_ values suggest that bicarbonate did not strongly stimulate canonical chemolithoautotrophic production, and instead reflect limited anaplerotic CO_2_ incorporation and/or cross-feeding on trace or sediment-derived organics.

Biomass-based specific growth rates were generally higher than substrate-based biomass generation rates. Previous studies reported similar discrepancies between biomass- and substrate-based estimates of biomass generation rates (Trembath-Reichert *et al.*, 2017; Morono *et al.*, 2011, 2020). This strongly suggests that the utilization of carbon and nitrogen compounds in the sediment other than the supplemented isotope-labeled substrates by active microbial communities is a common phenomenon for nutrient-starved subseafloor microbial communities. Nitrogen-based generation rates (^N^*μ*_S_) were detectable in many treatments and often exceeded ^C^*μ*_S_. Collectively, these results indicate that even when the carbon skeleton supply constrains biomass accrual, ammonium assimilation may broadly proceed.

### Microbial community patterns affected by substrate quality and oxygen availability

Microbial community compositions were profiled from FACS-sorted cells using 16S rRNA gene amplicons (contamination controls in Materials and Methods), and taxonomic profiles were obtained for all incubation conditions. By separating intact cells prior to DNA extraction, we reduced biases from extracellular/adsorbed DNA that may be severe in ultralow-biomass, fine-grained sediments. The starting sediment community (7X-1, day 0) was dominated by *Pseudomonadota* (formerly *Proteobacteria*) and *Bacillota* (formerly *Firmicutes*), consistent with oxic, oligotrophic marine sediments (Orsi, 2018; Hoshino *et al.*, 2020) (Fig. 4). Across incubations, community compositions showed temporal succession (10 → 30 → 75 d). By day 75, communities were dominated by *Gammaproteobacteria* across all treatments (88.2–97.5%; mean 93.2%). NMDS on CLR-transformed ASV compositions did not resolve discrete clustering by substrate or oxygen (Fig. 5 and Fig. S1). Therefore, the absence of discrete clustering suggests that samples followed broadly similar successional trajectories across treatments, shifting in similar directions through ordination space over time, whereas condition-specific enrichments were reflected mainly in differences in dominant lineages rather than in the clear separation of the overall community structure.

Amplicon tables are compositional, and changes in relative abundance may reflect either true changes in absolute abundance or taxonomic replacement. To partially distinguish these effects, we combined genus-level community profiles (relative abundance: Rel) with total cell concentrations (Cells) and calculated semi-quantitative, genus-specific abundance estimates (“Rel×Cells”; units follow the total cell concentration, cells cm^–3^). Genera reaching ≥20% relative abundance in at least one sample (including the initial sediment on day 0) were initially flagged as candidates (Fig. S2). Since Rel×Cells provides approximate estimates, values are interpreted as relative changes in the biomass contribution rather than exact absolute abundance. To avoid over-interpreting treatment-specific fluctuations given limited replications, we focused on five recurrently abundant genera—*Marinobacter*, *Alteromonas*, *Pseudomonas*, *Aeribacillus*, and *Geobacillus*—that capture the major successional and replacement patterns across incubations (hereafter “focal genera”; Fig. 6). Other genera exceeding the ≥20% threshold (including taxa unassigned at the genus level) are shown in Fig. S2.

Across incubations, *Bacillales* lineages (*e.g.*, *Aeribacillus* and *Geobacillus*) frequently showed pronounced early enrichment, typically by day 10, often without a corresponding increase in the total cell concentration. We interpreted these early *Bacillales* signals primarily as an incubation-associated response—likely the activation and relative enrichment of dormant populations triggered by physicochemical perturbations during sample recovery and incubation (*e.g.*, depressurization, mixing, and redox reorganization)—rather than as substrate-specific selection. Therefore, we distinguished this early incubation-associated response from subsequent substrate- and oxygen-dependent restructuring and biomass changes, rather than treating all early taxonomic shifts as substrate-specific selection.

Across amino acid-amended incubations, the five focal genera showed large changes in their estimated abundances (Rel×Cells) from early to late time points (Fig. 6). Under anoxic amino acid conditions, total cell concentrations remained nearly unchanged from days 0 to 10 (1.79×10^4^ to 1.99×10^4^ cells cm^–3^), while the relative contributions of the focal genera markedly shifted, indicating prominent taxonomic replacement without a net increase in the total cell concentration (a proxy for biomass). After day 10, total cell concentrations increased by nearly three orders of magnitude, reaching 1.71×10^7^ cells cm^–3^ by day 75, and the focal genera shifted from a mixed state on day 30 (including *Marinobacter*) to the strong late-stage dominance of *Pseudomonas* on day 75 (80.8%; estimated 1.38×10^7^ cells cm^–3^). Under oxic amino acid conditions, *Aeribacillus* was the most prominent focal genus on day 10 (33.8%; estimated 6.13×10^3^ cells cm^–3^), and *Geobacillus* was also elevated (14.3%; estimated 2.59×10^3^ cells cm^–3^), consistent with the early response of *Bacillales* described above rather than a substrate-specific response at this earliest stage. Total cell concentrations peaked on day 30 (1.94×10^6^ cells cm^–3^), when *Marinobacter* and *Alteromonas* were among the dominant focal genera, and then declined by day 75 (6.02×10^5^ cells cm^–3^), coinciding with the predominance of *Pseudomonas* (66.0%; estimated 3.97×10^5^ cells cm^–3^). Since *Pseudomonas* became dominant after total cell concentrations had already peaked or declined in some treatments, late-stage dominance appears to reflect a combination of growth, persistence, and replacement rather than monotonic biomass accumulation.

Bicarbonate-amended incubations recruited a narrower set of dominant responders among the focal genera (Fig. 6). Under anoxic bicarbonate conditions, *Marinobacter* became strongly dominant by day 75 (94.4%; estimated 5.71×10^6^ cells cm^–3^). Under oxic bicarbonate conditions, *Alteromonas* dominated by day 75 (85.3%; estimated 3.05×10^5^ cells cm^–3^). Although ^13^C-positive ROIs were detectable in bicarbonate incubations, the corresponding carbon-based generation rates (^C^μ_S_) remained low and were insufficient to fully account for the observed increases in total cell abundance. Therefore, bicarbonate-derived carbon was unlikely to be the dominant source of biomass under these conditions. The prominence of *Alteromonas* and *Marinobacter*, together with these low ^13^C-based generation rates, suggests that bicarbonate did not strongly stimulate canonical chemolithoautotrophic production. Instead, bicarbonate may have contributed through limited anaplerotic CO_2_ incorporation and/or physicochemical effects, such as buffering, while growth was likely supported partly by trace or sediment-derived organics.

Ammonium-only amendments produced the clearest redox-dependent contrast in population growth (cell concentrations) and community restructuring among the focal genera (Fig. 6). Under oxic NH_4_Cl-only conditions, *Geobacillus* strongly contributed on day 10 (21.1%; estimated 4.90×10^3^ cells cm^–3^), which is consistent with the early activation of *Bacillales* during incubation initiation. Between days 10 and 30, total cell concentrations increased from 2.32×10^4^ to 1.27×10^7^ cells cm^–3^ (~550-fold), which coincided with a shift to a *Marinobacter*-dominated community (79.0%; estimated 1.00×10^7^ cells cm^–3^). By day‍ ‍75, *Pseudomonas* increased and co-dominated with *Marinobacter* (58.5 and 37.6%, respectively), while total cell concentrations remained high (6.80×10^6^ cells cm^–3^). In contrast, under anoxic NH_4_Cl-only incubation conditions, total cell concentrations remained near the baseline until day 30 (1.79×10^4^–1.95×10^4^ cells cm^–3^) and increased only by day 75 (1.05×10^6^ cells cm^–3^), which coincided with the late-stage dominance of *Marinobacter* (78.9%; estimated 8.29×10^5^ cells cm^–3^). These paired oxic/anoxic NH_4_Cl-only incubations indicate that oxygen availability strongly affected the timing and magnitude of ammonium-stimulated population growth.

### Community restructuring, substrate assimilation, and incubation-associated effects

Whole-community ordination and single-cell NanoSIMS uptake data together indicate that amended substrates were not the sole drivers of biomass changes. NMDS did not resolve discrete clusters by substrate identity, whereas NanoSIMS showed that substrate-assimilating cells occurred across multiple amendment and redox conditions (Fig. 2 and 3). We calculated the fraction of analyzed cells exhibiting detectable ^13^C and/or ^15^N uptake for each incubation (Table 1). In anoxic amino acid incubations, the fraction of ^13^C-positive cells increased from 3.4% on day 10 to 69.3% on day 30 and 95.2% on day 75; ^15^N-positive fractions showed similar increases (10.3% → 69.3% → 95.2%). In contrast, under oxic amino acid conditions on day 10, one of the 110 ROIs exceeded the ^13^C uptake threshold, and one ROI exceeded the ^15^N uptake threshold despite the pronounced early enrichment of *Aeribacillus* and *Geobacillus* in the Rel×Cells profiles (representing 33.8 and 14.3% of the community, respectively, with estimated abundances of 6.13×10^3^ and 2.59×10^3^ cells cm^–3^). This decoupling supports the interpretation that‍ ‍early *Bacillales*-associated peaks primarily reflect incubation-associated physicochemical activation (*e.g.*, depressurization/redox reorganization) rather than the direct assimilation of the added labeled substrates. In bicarbonate incubations, ^13^C-positive ROIs were detected in a minority of cells, particularly on day 30; however, the associated carbon-based generation rates remained low. In contrast, ^15^N-positive ROIs became common by late time points in oxic and anoxic incubations (81.4 and 100%, respectively). These low carbon-based generation rates support the interpretation that bicarbonate acted mainly as a physicochemical modifier rather than driving broad chemo­litho­auto­trophic CO_2_ fixation detectable by ^13^C labeling. In NH_4_Cl-only incubations, ^15^N-positive fractions sharply increased under oxic conditions from 3.0% on day 10 to 98.9% on day 30, coinciding with the marked increase in total cell concentrations. In contrast, under anoxic conditions, uptake increased more gradually, reaching 85.5% on day 75. These uptake patterns, together with the weak separation by substrate identity in ordination space, indicate that amended substrates alone cannot explain the observed biomass changes. Rather, the incubations likely relaxed proximal constraints, such as ammonium availability and/or electron acceptor supply, enabling microbial populations to exploit endogenous sedimentary carbon pools. These results suggest that multiple constraints operate simultaneously, while the dominant *in situ* limitation is likely the extremely low energy flux resulting from limited supplies of both electron donors and acceptors.

## Conclusion

SAG subseafloor microbes are alive and responsive: we observed measurable ^13^C and ^15^N assimilation and positive biomass-based growth in multiple incubations, indicating that added substrates—and likely depressurization that loosened compacted fabrics and improved substrate accessibility—rekindled microbial activity. The imprint of long-term oxygen exposure on the community structure and substrate use was not prominent in our 0- to 75-d incubations. Across incubations, we observed a strong temporal succession, and community ordination did not show discrete clustering by oxygen regime. However, oxygen effects on biomass dynamics were treatment-specific (*e.g.*, strong stimulation in ammonium-only incubations). This contrasts with findings obtained from markedly older SPG sediments where activity was restricted to oxic conditions, suggesting that in relatively young (~6 Ma) SAG sediments, metabolic flexibility and the mixed use of endogenous resources are still retained.

Collectively, the present results show that, even after long-term burial in sediments, subseafloor microbial communities may mount rapid assimilation responses when substrates are supplied under both oxic and anoxic conditions. The quantitative linkage between isotope assimilation and biomass dynamics further supports that even in oligotrophic sediments, a fraction of the community may transition to a growth mode over short timescales once substrates become available.

## Acknowledgements

The samples used in this study were collected during IODP Expedition 390 conducted with the *R/V* JOIDES Resolution. We sincerely appreciate the work of the shipboard crew, operational team members, and shipboard scientists of IODP Expedition 390. The authors thank Takeshi Terada, Harumi Isshiki, and Kana Oka for their technical assistance in this study.

### Conflicts of Interest and Sources of Funding

The authors declare that there are no conflicts of interest. This research was supported by the Graduate School of Frontier Sciences, The University of Tokyo, through the Challenging New Area Doctoral Research Grant (Project No. C2406), JST SPRING Grant No. JPMJSP2108, and JSPS KAKENHI Grant Numbers 25KJ1107 (to MT) and 23H00154 (to YM). ERE was supported by NSF grants OCE-1326927 and OCE-2412279 while at Texas A&M University. Any opinions, findings, and conclusions or recommendations expressed in this material are those of the authors and do not necessarily reflect the views of the National Science Foundation.

### Data Availability

DNA sequence data are deposited in the DNA Data Bank of Japan (DDBJ) under the accession code DRA023882. The authors declare that the data supporting the results of this study are available within the manuscript and Supplementary Information.

## Citation

Takada, M., Ito, M., Wee, S. Y., Sylvan, J. B., Coggon, R. M., Estes, E. R., et al. (2026) Oxic Subseafloor Microbial Communities Retain Anabolic Capacity under both Oxic and Oxygen-depleted Conditions. *Microbes Environ ***41**: ME26008.

https://doi.org/10.1264/jsme2.ME26008

## Supplementary Material

Supplementary Material

## Figures and Tables

**Fig. 1.**
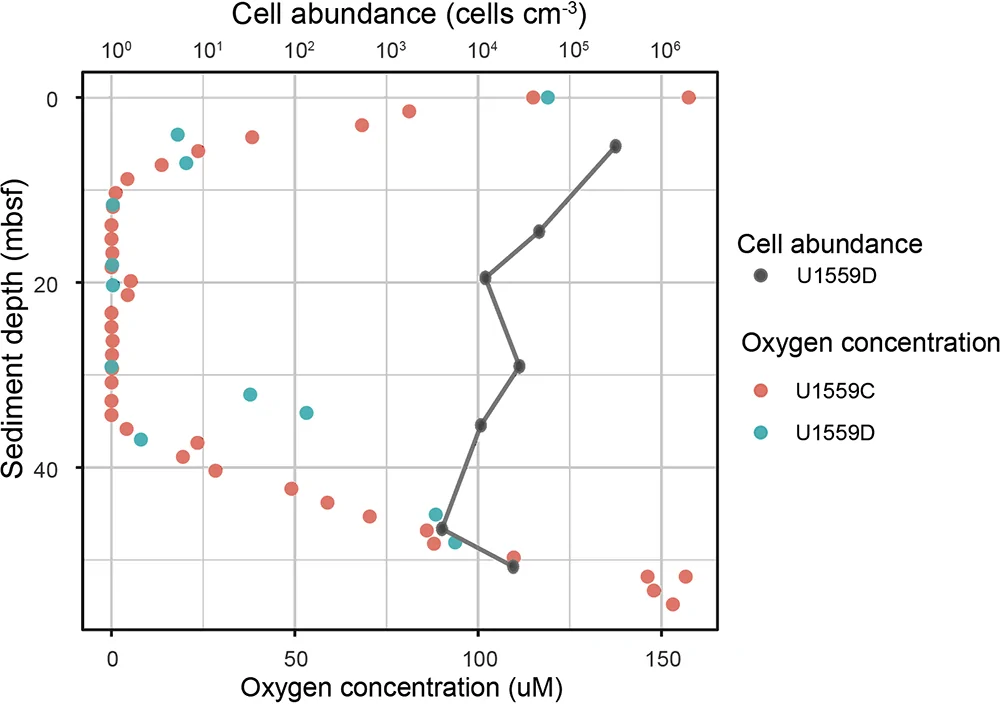
Depth profiles of log-scaled cell abundance and linear oxygen concentrations at U1559C/D. A combined plot of microbial cell abundance (top x-axis, log scale) and dissolved oxygen concentrations (bottom x-axis, μM, linear scale) of Site U1559 sediments (Holes C and D). Black line/points show the U1559D cell count profile; red (cyan) points indicate oxygen measurements from U1559C (U1559D). Depth increases downward (reversed y-axis).

**Fig. 2.**
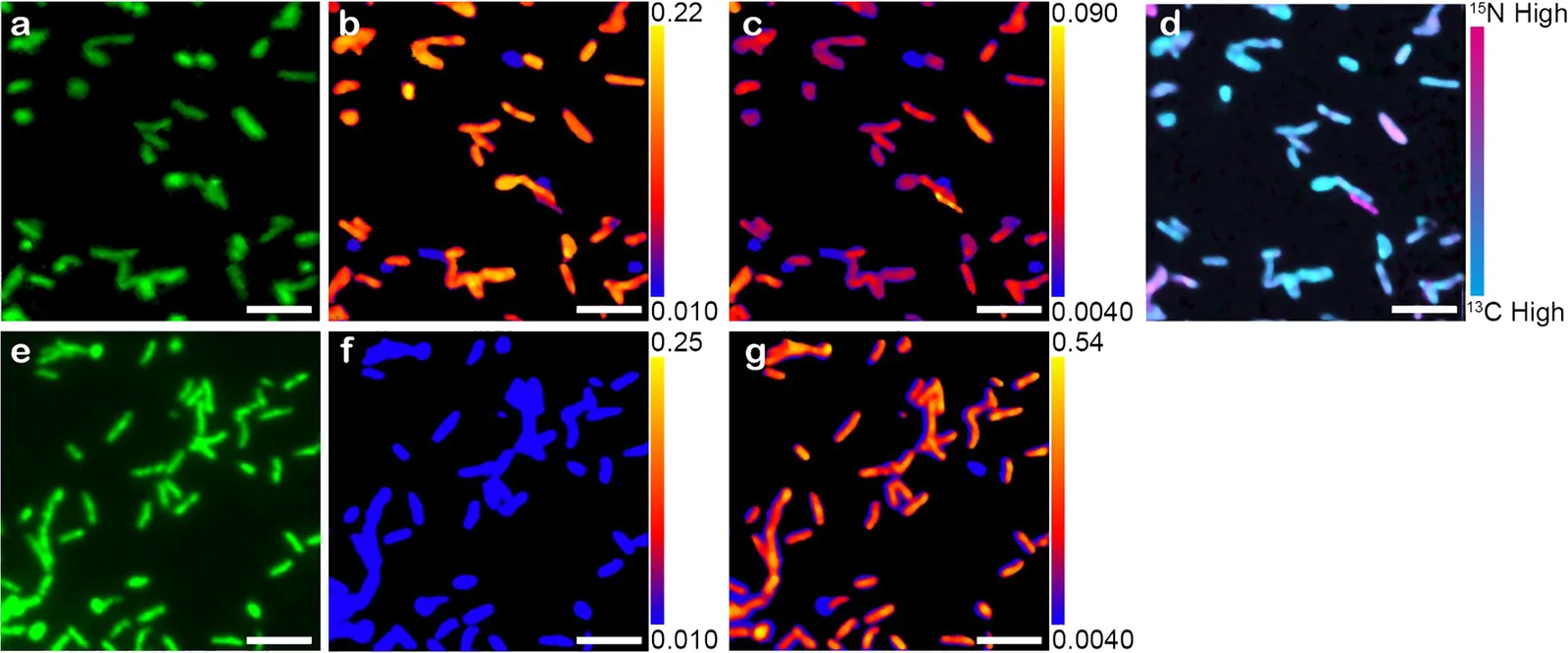
Magnitude and spatial distribution of ^13^C and ^15^N incorporation in representative microbial cells from Site U1559 (7X-1). Cells from incubations amended with a ^13^C-labeled amino acid mix plus ^15^N-ammonium are shown in (a, b, c, and d), and cells from incubations amended with ^15^N-ammonium only are shown in (e, f, and g). (a, e) SYBR Green I-stained cells under fluorescence microscopy. (b, f) Ratio images of ^13^C/^12^C. (c, g) Ratio images of ^15^N/^14^N acquired from the same regions as in (a) and (e), respectively, demonstrating the locations of ^13^C and ^15^N incorporation. Color scale ranges of the ratios are indicated by the values at the top and bottom of each color bar. The background membrane region identified from fluorescence images is excluded from ratio calculations and shown in black. (d) Overlay of (b) and (c) for the same field of view, visualizing ^13^C enrichment (cyan) and ^15^N enrichment (magenta). Bars represent 5 μm.

**Fig. 3.**
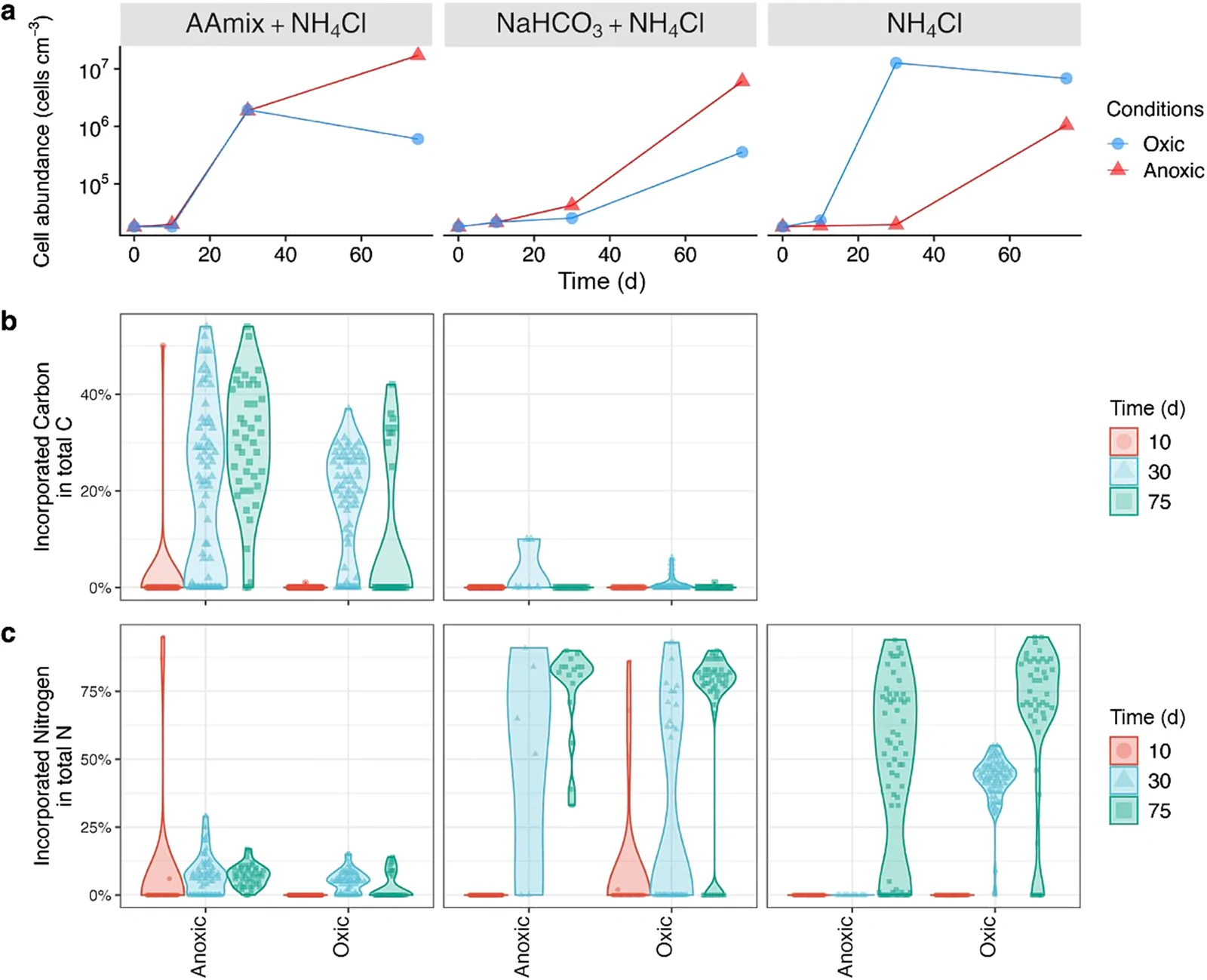
Microbial responses to substrate and incubation conditions. Plots are aligned vertically for each substrate treatment. (a) Cell abundances during incubations. Values on day 0 indicate cell densities in sediments prior to the incubation. Sediment samples amended with carbon substrates (amino acid mix [AAmix] or bicarbonate [NaHCO_3_]) were supplied with ammonium as the nitrogen source. The ammonium-only incubations (NH_4_Cl) received no added carbon substrate. In panel (a), colors and symbols distinguish redox conditions (oxic vs. anoxic). (b, c) Carbon (b) and nitrogen (c) substrate incorporation in single-cell ROIs identified by NanoSIMS. Incorporation was calculated as a fraction above natural abundance and plotted as violin plots (kernel density) overlaid with individual ROI values. In panels (b, c), colors and symbols indicate incubation times (10 d=red, 30 d=blue, 75 d=green), while oxic and anoxic conditions are shown along the x-axis.

**Fig. 4.**
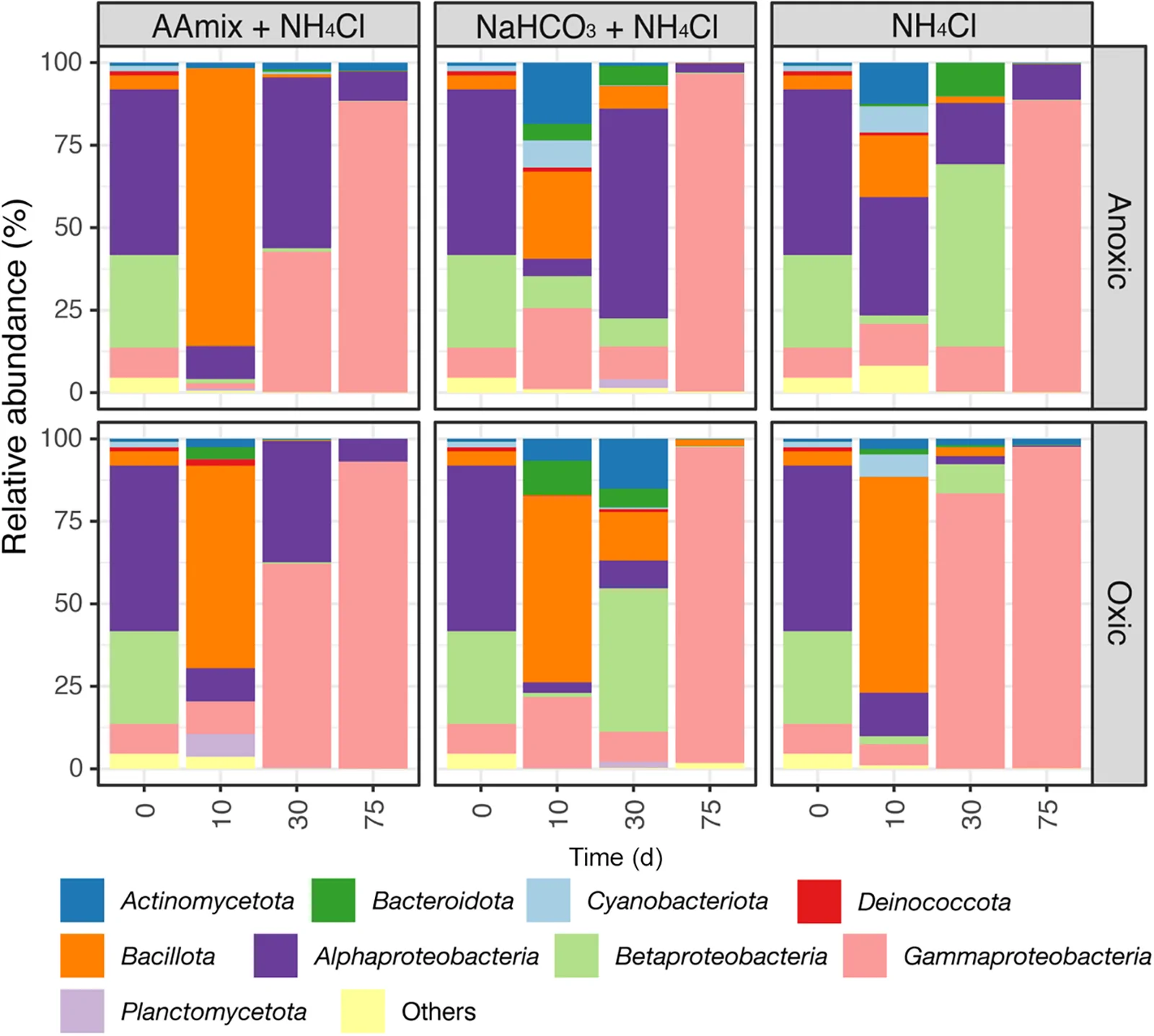
Shifts in the microbial community structure under different substrate and redox conditions. Relative abundances are shown as stacked bar plots of major phyla, with *Pseudomonadota* subdivided into *Alpha*-, *Beta*-, and *Gammaproteobacteria*. Each panel represents a substrate treatment (amino acid mix [AAmix]+NH_4_Cl, bicarbonate [NaHCO_3_]+NH_4_Cl, or ammonium-only [NH_4_Cl]), with anoxic conditions displayed in the top row and oxic conditions in the bottom row. The x-axis indicates incubation times (0, 10, 30, and 75 d), with less abundant groups pooled as “Others.”

**Fig. 5.**
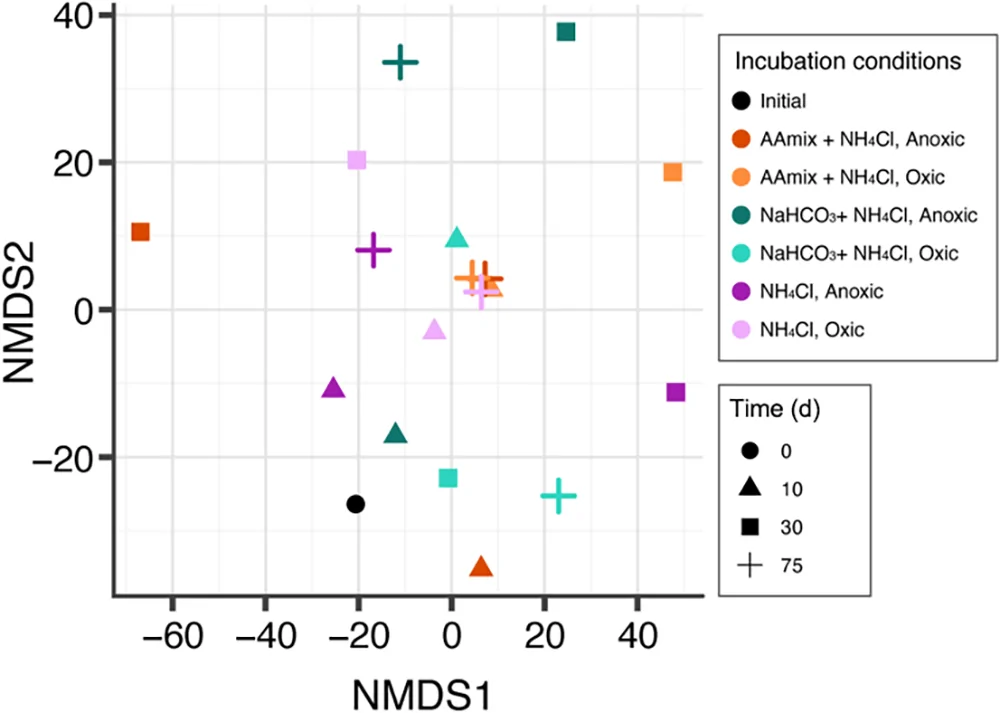
Non-metric multidimensional scaling (NMDS) ordination of CLR-transformed 16S rRNA gene-based microbial community compositions across incubation conditions. Colors indicate incubation conditions (substrate×redox), and symbols indicate incubation times.

**Fig. 6.**
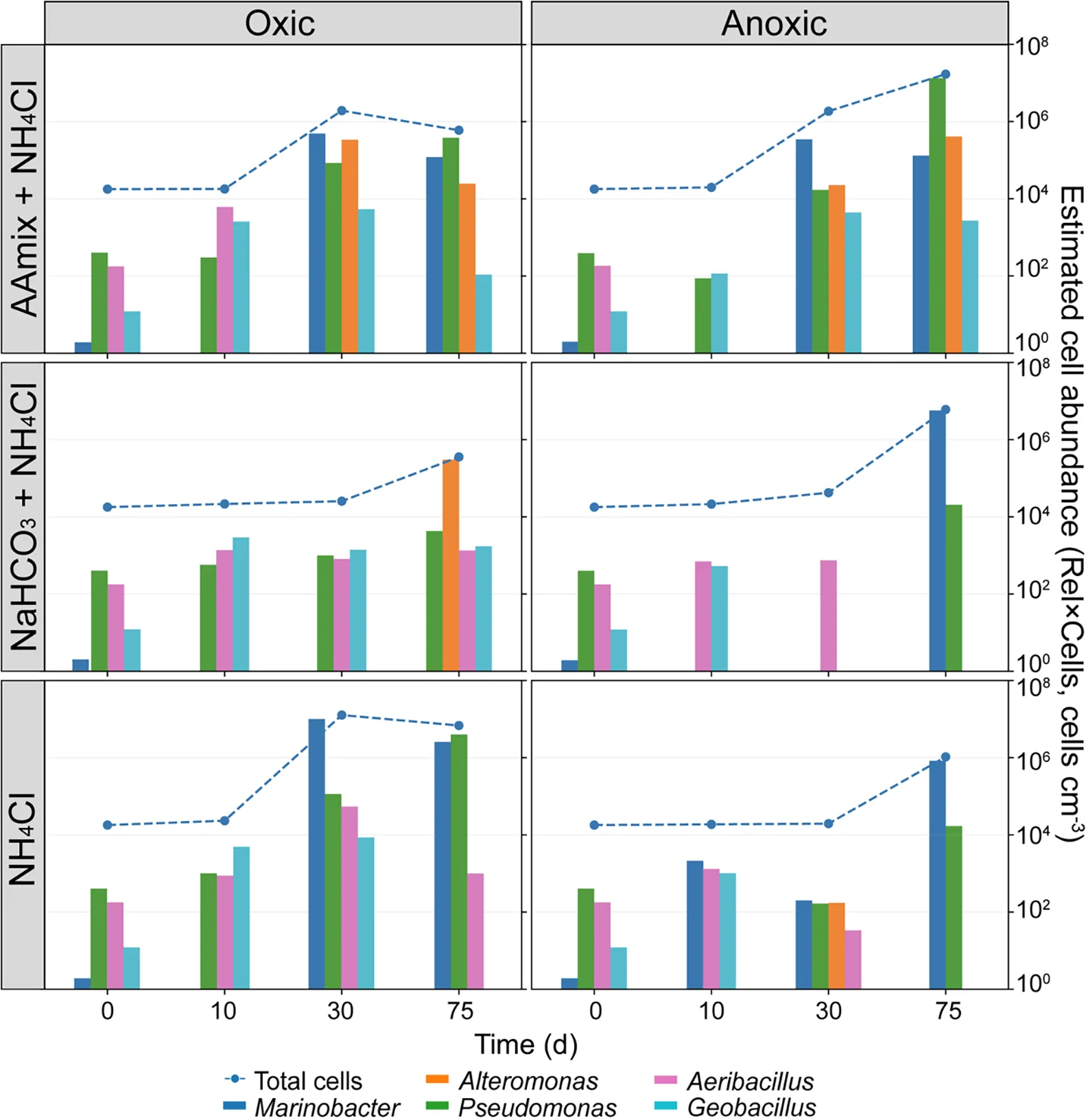
Cell-weighted (estimated absolute) genus dynamics during time-course incubations. Genus-level relative abundances obtained from 16S rRNA gene amplicon sequencing were scaled by total cell counts to estimate genus-specific abundances (Rel×Cells; grouped bars, log_10_ scale). The dashed line indicates the total cell counts for each sample on the same log scale. Panels show substrate amendments (amino acid mix [AAmix]+NH_4_Cl, bicarbonate [NaHCO_3_]+NH_4_Cl, or ammonium only [NH_4_Cl]) under oxic or anoxic conditions, sampled on 0, 10, 30, and 75 d; day 0 represents the initial sediment community and is shown in each panel as a reference. Only five genera (*Marinobacter*, *Alteromonas*, *Pseudomonas*, *Aeribacillus*, and *Geobacillus*) are shown here to highlight the major “replacement” patterns while maintaining interpretability; the expanded plot including all genera that reached ≥20% relative abundance in any sample, including the initial sediment, is provided in Fig. S2.

**Table 1.** Quantitative assessment of carbon and nitrogen assimilation and biomass growth under different substrate and redox conditions

Incubation condition	Incubation time (d)	Cell abundance (cells cm^–3^)	Substrate incorporation-based biomass generation rate (Carbon, ^C^μ_S_, d^–1^)	Substrate incorporation-based biomass generation rate (Nitrogen, ^N^μ_S_, d^–1^)	Biomass-based specific biomass changing rate (μ_B_, d^–1^)	Fraction of ^13^C–positive ROIs	Fraction of ^15^N–positive ROIs	Estimated original active fraction
AA mix+NH_4_Cl Anoxic	10	1.99×10^4^	0.072	0.175	0.010	3.4%	10.3%	0.67%
AA mix+NH_4_Cl Anoxic	30	1.87×10^6^	0.012	0.004	0.227	69.3%	69.3%	N/C
AA mix+NH_4_Cl Anoxic	75	1.71×10^7^	0.005	0.001	0.049	95.2%	95.2%	N/C
AA mix+NH_4_Cl Oxic	10	1.81×10^4^	0.001	0.000	0.001	0.9%	0.9%	0.087%
AA mix+NH_4_Cl Oxic	30	1.94×10^6^	0.009	0.002	0.234	80.6%	82.1%	N/C
AA mix+NH_4_Cl Oxic	75	6.02×10^5^	0.006	0.001	–0.026	29.0%	29.0%	N/C
NaHCO_3_+NH_4_Cl Anoxic	10	2.14×10^4^	N/C	N/C	0.017	0.0%	0.0%	N/C
NaHCO_3_+NH_4_Cl Anoxic	30	4.23×10^4^	0.004	0.052	0.034	33.3%	66.7%	21.44%
NaHCO_3_+NH_4_Cl Anoxic	75	6.05×10^6^	N/C	0.022	0.110	0.0%	100%	N/C
NaHCO_3_+NH_4_Cl Oxic	10	2.16×10^4^	N/C	0.066	0.019	0.0%	26.1%	11.06%
NaHCO_3_+NH_4_Cl Oxic	30	2.55×10^4^	0.001	0.046	0.008	25.7%	42.9%	18.91%
NaHCO_3_+NH_4_Cl Oxic	75	3.57×10^5^	0.000	0.023	0.059	2.3%	81.4%	N/C
NH_4_Cl Anoxic	10	1.87×10^4^	NA	N/C	0.004	NA	0.0%	N/C
NH_4_Cl Anoxic	30	1.95×10^4^	NA	N/C	0.002	NA	0.0%	N/C
NH_4_Cl Anoxic	75	1.05×10^6^	NA	0.013	0.089	NA	85.5%	N/C
NH_4_Cl Oxic	10	2.32×10^4^	NA	0.000	0.026	NA	3.0%	N/C
NH_4_Cl Oxic	30	1.27×10^7^	NA	0.019	0.315	NA	98.9%	N/C
NH_4_Cl Oxic	75	6.80×10^6^	NA	0.021	–0.014	NA	90.9%	N/C

NA (not applicable) denotes metrics that do not apply to the treatment (*e.g.*, tracer not added), whereas N/C (not calculable/not constrained) denotes metrics that apply, but were unable to be robustly computed from the data (*e.g.*, signal below detection/insufficient positives). Zero values indicate calculated values at or near zero within the assay’s resolution.

## References

[B1] Bar-On, Y.M., Phillips, R., and Milo, R. (2018) The biomass distribution on Earth. Proc Natl Acad Sci U S A 115: 6506–6511. 10.1073/pnas.1711842115 PMC6016768 29784790

[B2] Bolyen, E., Rideout, J.R., Dillon, M.R., Bokulich, N.A., Abnet, C.C., Al-Ghalith, G.A., et al. (2019) Reproducible, interactive, scalable and extensible microbiome data science using QIIME 2. Nat Biotechnol 37: 852–857. 10.1038/s41587-019-0209-9 PMC7015180 31341288

[B3] Bradley, J.A., Arndt, S., Amend, J.P., Burwicz, E., Dale, A.W., Egger, M., et al. (2020) Widespread energy limitation to life in global subseafloor sediments. Sci Adv 6: eaba0697. 10.1126/sciadv.aba0697 PMC7406382 32821818

[B4] Callahan, B.J., McMurdie, P.J., Rosen, M.J., Han, A.W., Johnson, A.J.A., and Holmes, S.P. (2016) DADA2: High-resolution sample inference from Illumina amplicon data. Nat Methods 13: 581–583. 10.1038/nmeth.3869 PMC4927377 27214047

[B5] Caporaso, J.G., Lauber, C.L., Walters, W.A., Berg-Lyons, D., Huntley, J., Fierer, N., et al. (2012) Ultra-high-throughput microbial community analysis on the Illumina HiSeq and MiSeq platforms. ISME J 6: 1621–1624. 10.1038/ismej.2012.8 PMC3400413 22402401

[B6] Coggon, R.M., Teagle, D.A.H., Sylvan, J.B., Reece, J., Estes, E.R., Williams, T.J., et al. (2024a) Expedition 390/393 methods. In *Proceedings of the International Ocean Discovery Program*. International Ocean Discovery Program. https://doi.org/10.14379/iodp.proc.390393.102.2024

[B8] Coggon, R.M., Teagle, D.A.H., Sylvan, J.B., Reece, J., Estes, E.R., Williams, T.J., et al. (2024b) Site U1559. In *Proceedings of the International Ocean Discovery Program*. International Ocean Discovery Program. 10.14379/iodp.proc.390393.109.2024

[B9] D’Hondt, S., Inagaki, F., Zarikian, C.A., Abrams, L.J., Dubois, N., Engelhardt, T., et al. (2015) Presence of oxygen and aerobic communities from sea floor to basement in deep-sea sediments. Nat Geosci 8: 299–304.

[B10] Dixon, P. (2003) VEGAN, a package of R functions for community ecology. J Veg Sci 14: 927–930.

[B11] Estes, E.R., Pockalny, R., D’Hondt, S., Inagaki, F., Morono, Y., Murray, R.W., et al. (2019) Persistent organic matter in oxic subseafloor sediment. Nat Geosci 12: 126–131.

[B12] Fuchs, G. (2011) Alternative pathways of carbon dioxide fixation: insights into the early evolution of life? Annu Rev Microbiol 65: 631–658. 10.1146/annurev-micro-090110-102801 21740227

[B13] Goethals, V., Vanwinsen, N., Mermans, F., De Gand, S., Boon, N., Van de Wiele, T., et al. (2025) An optimized flow cytometry sorting-sequencing workflow reduces storage, sorting and extraction bias in sorted microbial communities. bioRxiv. https://doi.org/10.1101/2025.11.13.688239

[B14] Gormanns, P., Reckow, S., Poczatek, J.C., Turck, C.W., and Lechene, C. (2012) Segmentation of multi-isotope imaging mass spectrometry data for semi-automatic detection of regions of interest. PLoS One 7: e30576. 10.1371/journal.pone.0030576 PMC3276494 22347386

[B15] Hoshino, T., Doi, H., Uramoto, G.-I., Wörmer, L., Adhikari, R.R., Xiao, N., et al. (2020) Global diversity of microbial communities in marine sediment. Proc Natl Acad Sci U S A 117: 27587–27597. 10.1073/pnas.1919139117 PMC7959581 33077589

[B16] Hügler, M., and Sievert, S.M. (2011) Beyond the Calvin cycle: autotrophic carbon fixation in the ocean. Ann Rev Mar Sci 3: 261–289. 10.1146/annurev-marine-120709-142712 21329206

[B17] Jalaluddin, F.M., Ahmerkamp, S., Marchant, H.K., Meyer, V., Koren, K., and Kuypers, M.M.M. (2025) Microenvironments on individual sand grains enhance nitrogen loss in coastal sediments. Sci Rep 15: 16384. 10.1038/s41598-025-00755-3 PMC12066709 40350486

[B18] Kallmeyer, J., Pockalny, R., Adhikari, R.R., Smith, D.C., and D’Hondt, S. (2012) Global distribution of microbial abundance and biomass in subseafloor sediment. Proc Natl Acad Sci U S A 109: 16213–16216. 10.1073/pnas.1203849109 PMC3479597 22927371

[B19] Lever, M.A., Rogers, K.L., Lloyd, K.G., Overmann, J., Schink, B., Thauer, R.K., et al. (2015) Life under extreme energy limitation: a synthesis of laboratory- and field-based investigations. FEMS Microbiol Rev 39: 688–728. 10.1093/femsre/fuv020 25994609

[B20] Lipp, J.S., Morono, Y., Inagaki, F., and Hinrichs, K.-U. (2008) Significant contribution of Archaea to extant biomass in marine subsurface sediments. Nature 454: 991–994. 10.1038/nature07174 18641632

[B21] Magnabosco, C., Lin, L.-H., Dong, H., Bomberg, M., Ghiorse, W., Stan-Lotter, H., et al. (2018) The biomass and biodiversity of the continental subsurface. Nat Geosci 11: 707–717.

[B22] McMurdie, P.J., and Holmes, S. (2013) phyloseq: an R package for reproducible interactive anal­ysis and graphics of microbiome census data. PLoS One 8: e61217. 10.1371/journal.pone.0061217 PMC3632530 23630581

[B23] Morono, Y., Terada, T., Masui, N., and Inagaki, F. (2009) Discriminative detection and enumeration of microbial life in marine subsurface sediments. ISME J 3: 503–511. 10.1038/ismej.2009.1 19212428

[B24] Morono, Y., and Inagaki, F. (2010) Automatic slide-loader fluorescence microscope for discriminative enumeration of subseafloor life. Sci Drill 9: 32–36.

[B25] Morono, Y., Terada, T., Nishizawa, M., Ito, M., Hillion, F., Takahata, N., et al. (2011) Carbon and nitrogen assimilation in deep subseafloor microbial cells. Proc Natl Acad Sci U S A 108: 18295–18300. 10.1073/pnas.1107763108 PMC3215001 21987801

[B26] Morono, Y., Terada, T., Kallmeyer, J., and Inagaki, F. (2013) An improved cell separation technique for marine subsurface sediments: applications for high-throughput anal­ysis using flow cytometry and cell sorting: Counting and sorting cells from marine sediments. Environ Microbiol 15: 2841–2849. 10.1111/1462-2920.12153 PMC3910163 23731283

[B27] Morono, Y., Terada, T., Hoshino, T., and Inagaki, F. (2014) Hot-alkaline DNA extraction method for deep-subseafloor archaeal communities. Appl Environ Microbiol 80: 1985–1994. 10.1128/AEM.04150-13 PMC3957647 24441163

[B28] Morono, Y., Ito, M., Hoshino, T., Terada, T., Hori, T., Ikehara, M., et al. (2020) Aerobic microbial life persists in oxic marine sediment as old as 101.5 million years. Nat Commun 11: 3626. 10.1038/s41467-020-17330-1 PMC7387439 32724059

[B29] Orsi, W.D., Coolen, M.J.L., Wuchter, C., He, L., More, K.D., Irigoien, X., et al. (2017) Climate oscillations reflected within the microbiome of Arabian Sea sediments. Sci Rep 7: 6040. 10.1038/s41598-017-05590-9 PMC5519670 28729646

[B30] Orsi, W.D. (2018) Ecology and evolution of seafloor and subseafloor microbial communities. Nat Rev Microbiol 16: 671–683. 10.1038/s41579-018-0046-8 29967420

[B31] Schindelin, J., Arganda-Carreras, I., Frise, E., Kaynig, V., Longair, M., Pietzsch, T., et al. (2012) Fiji: an open-source platform for biological-image analysis. Nat Methods 9: 676–682. 10.1038/nmeth.2019 PMC3855844 22743772

[B32] Schneider, C.A., Rasband, W.S., and Eliceiri, K.W. (2012) NIH Image to ImageJ: 25 years of image analysis. Nat Methods 9: 671–675. 10.1038/nmeth.2089 PMC5554542 22930834

[B33] Tengberg, A., Hovdenes, J., Andersson, H.J., Brocandel, O., Diaz, R., Hebert, D., et al. (2006) Evaluation of a lifetime-based optode to measure oxygen in aquatic systems. Limnol Oceanogr Methods 4: 7–17.

[B34] Trembath-Reichert, E., Morono, Y., Ijiri, A., Hoshino, T., Dawson, K.S., Inagaki, F., et al. (2017) Methyl-compound use and slow growth characterize microbial life in 2-km-deep subseafloor coal and shale beds. Proc Natl Acad Sci U S A 114: E9206–E9215. 10.1073/pnas.1707525114 PMC5676895 29078310

[B35] Whitman, W.B., Coleman, D.C., and Wiebe, W.J. (1998) Prokaryotes: the unseen majority. Proc Natl Acad Sci U S A 95: 6578–6583. 10.1073/pnas.95.12.6578 PMC33863 9618454

