# Supplementary material for: Oxic Subseafloor Microbial Communities Retain Anabolic Capacity under both Oxic and Oxygen-depleted Conditions
Source: Microbes Environ. 2026 Jul 30;41(3):ME26008. doi: 10.1264/jsme2.ME26008 (PMC13659347; doi:10.1264/jsme2.ME26008)
Supplement: Supplementary file 1 [file 41_26008_s1.pdf]

Supplementary Materials for

Oxic Subseafloor Microbial Communities Retain Anabolic Capacity under both Oxic and Oxygen-depleted Conditions

Mako Takada[1,2,3], Motoo Ito[3], Shu Ying Wee[4], Jason B. Sylvan[5], Rosalind M. Coggon[6], Emily R. Estes[5,7], William P. Gilhooly III[8], Yi Wang[9], Masataka Aizawa[10], Susumu Yoshizawa[1,2] and Yuki Morono[3*]

1 Atmosphere and Ocean Research Institute, The University of Tokyo, Japan

2 Graduate School of Frontier Sciences, The University of Tokyo, Japan

3 Kochi Institute for Core Sample Research, Japan Agency for Marine-Earth Science and Technology (JAMSTEC), Japan

4 University of Southern California, USA

5 Texas A&M University, USA

6 University of Southampton, United Kingdom

7 Now at National Science Foundation, Alexandria, VA, USA

8 Indiana University Indianapolis, USA

9 Tulane University, USA

10 Hokkaido University of Education, Japan

* Corresponding Author. Email: morono@jamstec.go.jp; Tel +81-88-878-2273

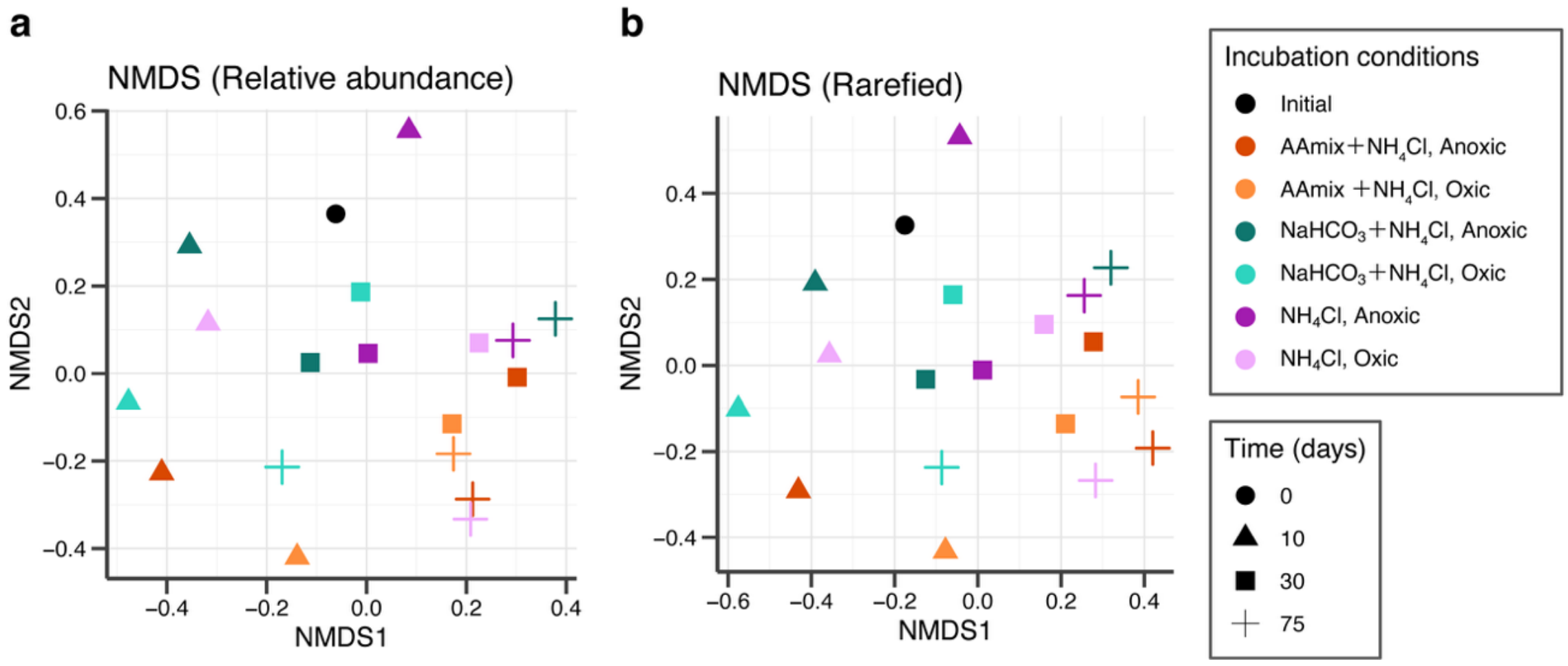

Supplementary Figure S1 | Non-metric multidimensional scaling (NMDS) ordination of Bray–Curtis dissimilarities calculated from relative-abundance and rarefied 16S rRNA gene amplicon profiles of South Atlantic Gyre sediment incubations. Colors indicate incubation condition (substrate × redox), and symbols indicate incubation time. Panels show NMDS results based on relative abundance (a) and rarefied counts (b). AAmix denotes the amino acid mix.

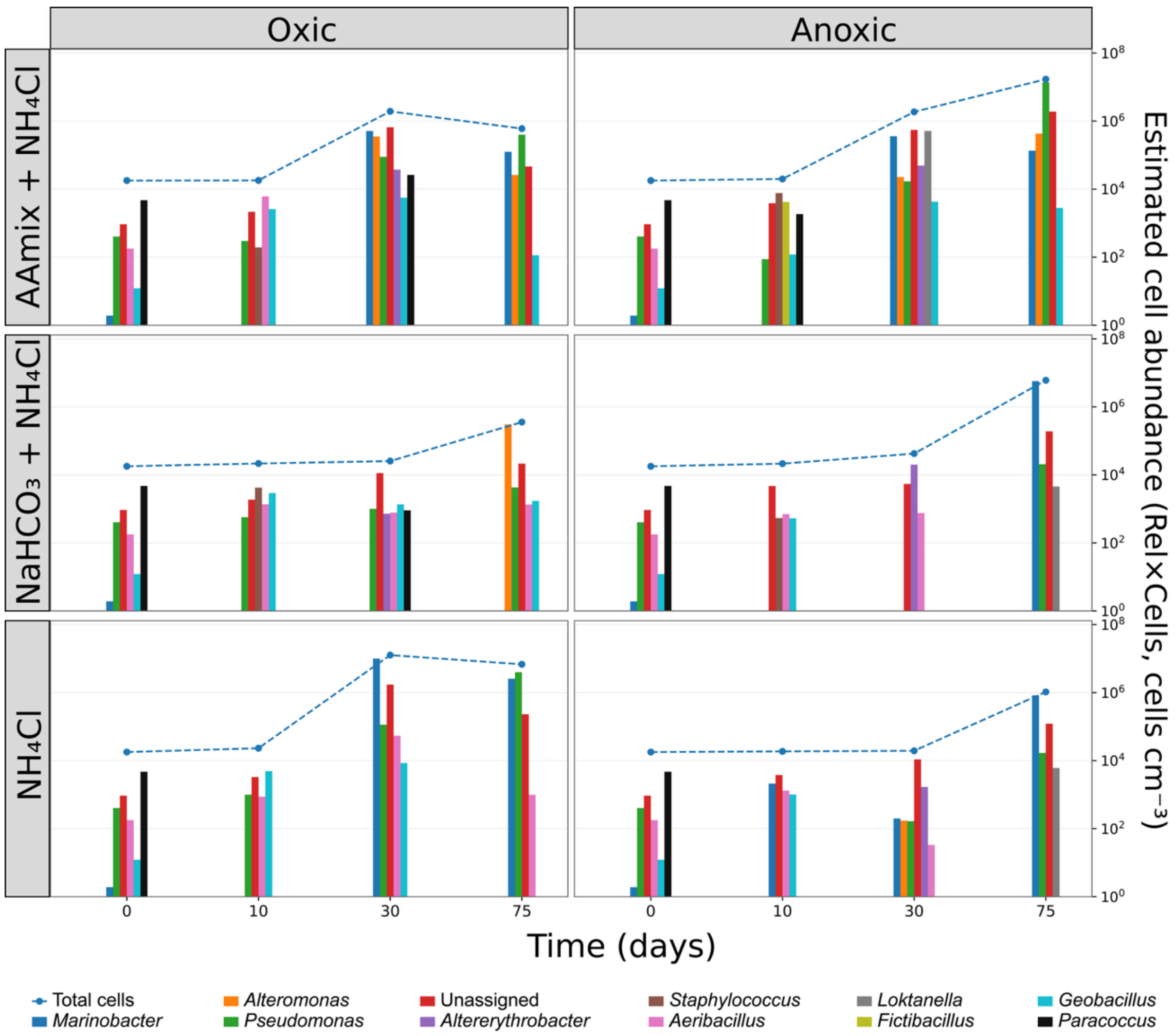

Supplementary Figure S2 | Estimated absolute (cell-weighted) abundances of the 10 dominant genera and the genus-unassigned category across incubation time under each substrate × redox condition. Genus-level relative abundances from 16S rRNA gene amplicon profiles of cells sorted by fluorescence-activated cell sorting (FACS) were converted to estimated absolute abundances by multiplying by the total cell concentration for each sample (Rel × Cells; bars; log scale; cells cm^{-3}). The dashed line indicates the total cell concentration (cells cm^{-3}). Time points (Days 0, 10, 30, and 75) are shown at equal spacing for clarity. Day 0 represents the same initial sediment community and is repeated in each panel as a common reference. The 10 genera and the genus-unassigned category were selected in a data-driven manner as those reaching a relative abundance of ≥20% in at least one sample, including the initial sediment at Day 0: *Marinobacter*, *Alteromonas*, *Pseudomonas*, *Altererythrobacter*, *Loktanella*, *Aeribacillus*, *Geobacillus*, *Fictibacillus*, *Staphylococcus*, *Paracoccus*, and "Unassigned" (reads not classified to the genus level). This extended panel complements the main-text figure by displaying the full ≥20% set.
